# Role of bacterial pathogens in microbial ecological networks in hydroponic plants

**DOI:** 10.3389/fpls.2024.1403226

**Published:** 2024-09-03

**Authors:** Wenyi Liu, Zhihua Zhang, Bin Zhang, Yi Zhu, Chongwen Zhu, Chaoyong Chen, Fangxu Zhang, Feng Liu, Jixiang Ai, Wei Wang, Wuyuan Kong, Haoming Xiang, Weifeng Wang, Daoxin Gong, Delong Meng, Li Zhu

**Affiliations:** ^1^ College of Environment and Ecology, Hunan Agricultural University, Changsha, China; ^2^ School of Minerals Processing and Bioengineering, Central South University, Changsha, China; ^3^ Changde Tobacco Company of Hunan Province, Changde, China; ^4^ College of Chemistry and Bioengineering, Hunan University of Science and Engineering, Yongzhou, China

**Keywords:** *Pseudomonas*, wildfire disease, pathogen invasion, molecular ecological network, compartment niches, plant-microbe interactions

## Abstract

Plant-associated microbial communities are crucial for plant growth and health. However, assembly mechanisms of microbial communities and microbial interaction patterns remain elusive across vary degrees of pathogen-induced diseases. By using 16S rRNA high-throughput sequencing technology, we investigated the impact of wildfire disease on the microbial composition and interaction network in plant three different compartments. The results showed that pathogen infection significantly affect the phyllosphere and rhizosphere microbial community. We found that the primary sources of microbial communities in healthy and mildly infected plants were from the phyllosphere and hydroponic solution community. Mutual exchanges between phyllosphere and rhizosphere communities were observed, but microbial species migration from the leaf to the root was rarely observed in severely infected plants. Moreover, wildfire disease reduced the diversity and network complexity of plant microbial communities. Interactions among pathogenic bacterial members suggested that Caulobacter and Bosea might be crucial “pathogen antagonists” inhibiting the spread of wildfire disease. Our study provides deep insights into plant pathoecology, which is helpful for the development of novel strategies for phyllosphere disease prediction or prevention.

## 1 Introduction

Pseudomonas is a common pathogenic bacterium found on Solanaceae crops (Höfte and De Vos, 2007). *Pseudomonas syringae* is a Gram-negative bacterium, and wildfire disease caused by *Pseudomonas syringae* is a major destructive bacterial leaf disease worldwide (Lucas, 1958). It affects various plants, including tobacco, tomato, citrus, and beans (Morris et al., 2007; O'Brien et al., 2011), causing significant crop losses globally (Wang et al., 2013). Several studies suggest that microbes residing on plants without inducing disease may also contribute to host resistance against pathogens (Chapelle et al., 2016; Mendes et al., 2018). Therefore, a better understanding of the changes in the plant microbial community during the disease process is necessary for developing biological control strategies to manage wildfire disease.

Plants harbor complex bacterial communities in various plant parts, each exerting distinct roles (Dong et al., 2019). Diverse microorganisms inhabit different plant compartments, including roots, stems, leaves, flowers, and fruits (Lindow and Brandl, 2003; Hacquard et al., 2015). The phyllosphere, residing on plant surfaces, hosts numerous potentially beneficial, pathogenic, or antagonistic microorganisms (bacteria, fungi, and viruses), with the composition and diversity of leaf surface microbial communities influenced by plant diseases (Aleklett et al., 2014). The root system constitutes a crucial area for plant-microbe interactions (Edwards et al., 2015). Bacteria in the rhizosphere can induce or suppress diseases, produce plant growth regulators and other biologically active substances, or influence plant productivity by modulating the availability of nutrients and toxic elements (Saeed et al., 2021). The hydroponic solution for hydroponic plants consists primarily of inorganic ions providing essential elements for higher plants, obtained from the growth medium (Trejo-Téllez and Gómez-Merino, 2012). Despite prior research predominantly focusing on individual plant parts in wildfire disease, without exploration in a unified context involving all three compartments at varying severity levels, the shaping of microbial community assemblies and symbiotic patterns across the rhizosphere, phyllosphere, and endosphere remains largely unknown.

Infection by pathogens can have a significant impact on the resident microbial community. The pathogen may disrupt interactions among plant microbiota, leading to the restructuring of microbial communities (Van der Putten et al., 2007). However, current understanding of how pathogen infection in Solanaceae crops induces changes in microbial communities remains limited. Recent studies have suggested that during the invasion of bacterial wilt, the endophytic community was significantly affected by the invasion of the pathogen. Furthermore, interactions among pathogenic members suggested a positive correlation between pathogenic members and the presence of Delftia, Stenotrophomonas, and Bacillus in the infected plant roots (Hu et al., 2020). A recent study proposed that the pathogen Xanthomonas causing bacterial leaf spot disease in lettuce showed a significant positive correlation with the genus Alkanindiges, but exhibited negative correlations with the genera Bacillus, Erwinia, and Pantoea (Rastogi et al., 2012) Previous studies have shown that members of Botryosphaeria, Paraphoma, and Plectosphaerella in the phyllosphere fungal community of Solanaceae crops infected with leaf spot disease may act as crucial “pathogen facilitators” to exacerbate the severity of brown spot disease. Conversely, genera Pleospora and Ochrocladosporium could serve as significant “pathogen antagonists” to inhibit the expansion of pathogenic Alternaria (Tao et al., 2021). However, there have been limited studies investigating the interactions between microbial communities in plant leaves, roots, hydroponic solutions, and the pathogenic bacterium Pseudomonas. These relationships between microbial communities and Pseudomonas might uncover the roles played by various microbes in either inhibiting or promoting wildfire disease.

The objectives of this study were to (i) characterize the diversity and structure of phyllosphere, root and hydroponic solution bacteria communities at different disease severities of wildfire disease;(ii)The source of microbial migration from hydroponic solutions to endophytic communities under the influence of wildfire disease.;(iii) use association networks to examine the frequency of interactions within microbial communities associated with the disease severities and compartments of Solanceae crops and analyze the interactions between the pathogen and other microbiota through network analysis.

## 2 Materials and methods

### 2.1 Sample collection and processing

All 27 samples were collected in June 2022(the early stage of wildfire disease) from Kunming City, Yunnan Province, China (24°23’N, 102°10’E), plants without any symptoms of wildfire disease were classified as healthy plants, while those showing symptoms were classified as diseased plants. The infection levels ranging from 3 to 9, and the severity classification standards for bacterial wildfire disease are based on the pest classification and survey methods (GB/T 23222–2008), P.R. China. Three plants were randomly selected from each category of the healthy, mildly infected, and severely infected plants for sampling. Subsequently, samples were collected from the phyllosphere, root and hydroponic solution of the same plants. All samples were transported to the laboratory on dry ice and labeled as H (healthy), S (slight infected), and I (severely infected). Each sample was a composite sample mixed from three subsamples taken from the same plant.

Gently stir the hydroponic solution to ensure even mixing. Using a sterile pipette, draw 50 mL of the hydroponic solution and store the obtained liquid at -80°C for subsequent DNA extraction. Roots and stems are washed separately with 75% ethanol, 2.5% sodium hypochlorite, and sterile water. Subsequently, the roots and stems are cut into small pieces and stored at -80°C for DNA extraction.

### 2.2 DNA extraction and amplicon sequencing

The DNA extraction was performed using the E.Z.N.A.^®^Soil DNA Kit (Omega, USA) according to the instructions, with 1.0 g of phyllosphere and root microbiota for DNA extraction. Additionally, the E.Z.N.A.^®^Water DNA Kit (Omega, USA)was employed to extract DNA from 5.00 ml of hydroponic solution. The 16S rRNA variable region (V3+V4) was amplified by PCR using primers 338F (5’-ACTCCTACGGGAGGCAGCAG-3’) and 806R (5’-GGACTACHVGGGTWTCTAAT-3’) (Srinivasan et al., 2012). The PCR conditions consisted of a 50 μL reaction system including 1.5 μL dNTP mix, 0.5 μL TaqDNA enzyme (Takara, Beijing, China), 5 μL 10× PCR buffer, 1.5 μL of 10 μM forward and reverse primers, and 20~30 ng of template. The thermal cycling operations was defined as: 94 for 1 minute, 30 cycles of 94 for 20 seconds, 57 for 25 seconds, 68 for 45 seconds, and a final elongation at 72 for 10 minutes, and finally stored at 4 . The PCR products were detected by 2% agarose gel electrophoresis, and the target fragments were recovered using the AxyPrep PCR Cleanup Kit. The purified PCR products were quantified using the Quant-iT PicoGreen dsDNA Assay Kit on the Qubit fluorescence quantification system. After gradient dilution of each qualified on-machine sequencing library (the Index sequence is not repeatable), they were mixed in corresponding proportions according to the required sequencing amount and denatured by NaOH into single strands for on-machine sequencing. These samples were performed to 2×300bp paired-end sequencing using the MiSeq sequencer from Hangzhou Lianchuan Biotechnology Co., Ltd. with the corresponding MiSeq Reagent Kit.

### 2.3 Sequence data preprocessing and statistical analysis

The raw gene sequences were analyzed using the QIIME 2 (Bolyen et al., 2019). Initially, DADA2 (Callahan et al., 2016) was employed to filter low-quality sequences (truncQ = 2, maxN = 0, maxEE = c(3, 5)). Then, sequences were clustered into Operational Taxonomic Units (OTUs) at a 97% similarity level, generating an operational taxonomic unit table. Subsequently, OTUs were taxonomically classified by comparing them with the silva-138-99-nb-classifier (Yilmaz et al., 2014) prokaryotic organism database. A systematic evolutionary tree was constructed using representative sequences of OTUs, and species annotations were performed.

All statistical analyses and computations were conducted using the R platform (version 4.1.1). The microeco 0.5.1 package (Liu et al., 2021) was employed for ecological data statistics and visualization of bacterial communities. The ggplot2 (Wickham et al., 2016) package was utilized to generate species abundance plots, and the α-diversity index (Shannon index) of bacterial communities was calculated. Kruskal-Wallis rank-sum tests and multiple comparisons of variance were performed to examine inter-group differences in alpha diversity of bacterial communities. Principal Coordinate Analysis (PCoA) was applied for dimensionality reduction of microbial communities. The Bray-Curtis distance was computed to assess community beta diversity. The Adonis (Dixon, 2003) function was employed for permutation multivariate analysis of variance (PERMANOVA) statistical tests, evaluating the relative contributions of different factors to community dissimilarities. The Linear Discriminant Analysis Effect Size (LEfSe) was used to identify statistically different biomarkers at various taxonomic levels between groups (Segata et al., 2011). The SourceTracker model was utilized to estimate the sources of bacterial communities in different parts of healthy and wildfire-infected plants.

### 2.4 Network construction

To elucidate the interactions of plant microbial communities during wildfire disease invasion, we constructed Molecular Ecological Networks (MENs) based on Sparcc correlation coefficients (P < 0.05). Greedy module optimization methods were employed for module separation. The optimal correlation coefficient threshold was determined using Random Matrix Theory (RMT). Additionally, a consistent threshold was selected to generate networks for comparison under the same conditions (Deng et al., 2012). The networks of phyllosphere, root, and hydroponic solution communities were analyzed using the mentioned approach. Subnetworks of interactions between pathogens and other microbial members were constructed in phyllosphere and root samples of slight infected plants. All networks were visualized using Gephi (Bastian et al., 2009). The calculated topological characteristics of bacterial networks included symbiotic (positive) and exclusion (negative) correlation numbers, average path length, network diameter, average clustering coefficient, average connectivity, and modularity.

## 3 Results

### 3.1 bacterial community structures diversity and composition of healthy and different disease severities infected samples

**Figure 1:**
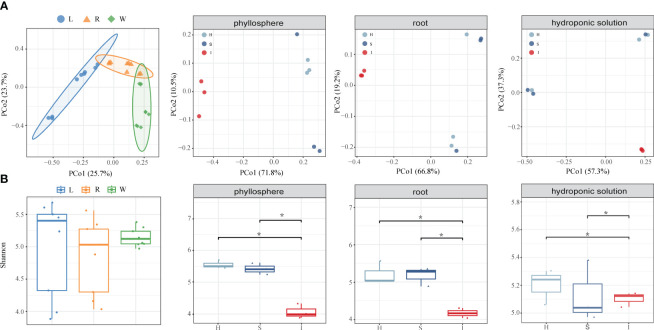
Assembly of phyllosphere, root and hydroponic solution bacterial communities. **(A)** Principal coordinate analysis (PCoA) of Bray–Curtis dissimilarity matrices showing the effects of compartment niches and bacterial wildfire disease on the community structure of the phyllosphere, root and hydroponic solution bacterial microbiomes. **(B)** Shannon diversity index of compartment niches and bacterial wildfire disease impact on the community structure of the phyllosphere, root, and hydroponic solution bacterial microbiomes, indicating significant differences (*P < 0.05).

5403 OTUs were obtained from 27 bacterial samples. PERMANOVA analysis (**Supplementary Table 1**) suggested that the variation in bacteria was mainly influenced by compartment niches (R2 = 38.97%, P=0.001) and disease severity (R2 = 9.78%, P=0.02). PCoA of Bray–Curtis distance revealed that the phyllosphere, root and hydroponic solution formed three distinct clusters regardless of plant disease severity. The PCoA of each compartment microbiome indicated significant differences in the community structures of the phyllosphere, root and hydroponic due to wildfire disease. Compared to its effects on the bacterial communities in the phyllosphere and roots, the impact of wildfire disease on the bacterial community in the hydroponic solution was less pronounced (**Figure 1**; **Supplementary Table 2**; phyllosphere, root and hydroponic solution:P<0.05, P<0.005, P<0.1).

Bacterial communities in all compartment niches had a similar Shannon diversity index. The bacterial alpha diversity in each compartment niches was affected by disease severity (ANOVA; P < 0.05). Shannon diversity gradually decreased from healthy to slight to severe, indicating that diversity of bacteria communities did not change significantly in the early stage of disease infection but dramatically decreased in cases of severe infected disease. The variation in bacterial community diversity was more pronounced in the phyllosphere and root compartments compared to the hydroponic solution.

**Figure 2:**
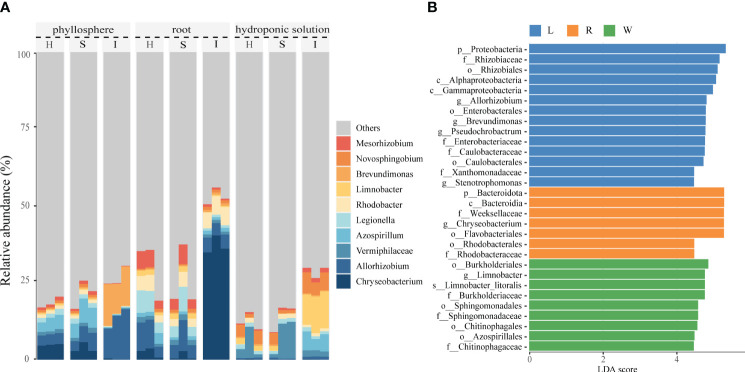
Comparison of phyllosphere, root and hydroponic solution community structures in healthy and infected samples **(A)** Relative abundance of bacterial genera of top 10 most dominant OTUs in healthy and infected phyllosphere, root and hydroponic solution. **(B)** The linear discriminant analysis effect size (LEfSe) analysis at species level of bacterial communities (with LDA score >3.1 and p < 0.05) among healthy and infected phyllosphere, root and hydroponic solution.

All OTUs were classified into 36 phyla. The top 10 most dominant OTUs (≥1.0% relative abundance) in the samples are shown in **Supplementary Figure 1**.The predominant phylum across all three compartment was Proteobacteria, accounting for no less than 38% of the relative abundance, followed by Bacteroidota, Firmicutes, and Actinobacteriota. In comparison to hydroponics, the proportions of Bacteroidota, Proteobacteria, and Firmicutes significantly increased on phyllosphere and roots, while Verrucomicrobiota, Planctomycetota, and Dependentiae were largely depleted. The compositional differences among the three compartments were more pronounced at the genus level, with all OTUs classified into 709 genera. The top 10 most abundant OTUs (≥1.0% relative abundance) were illustrated in **Figure 2A**. The compositions of phyllosphere, roots, and hydroponic solution were generally similar, with dominant genera including Chryseobacterium, Azospirillum, Vermiphilaceae, Allorhizobium, Brevundimonas, and Arcicella. The invasion of pathogenic wildfire disease altered the relative abundance of these genera in different compartment, with phyllosphere and roots experiencing more significant impacts than the hydroponic solution. In the phyllosphere, as the disease severity increased, the relative abundance of Chryseobacterium, Azospirillum, and Vermiphilaceae decreased, while Allorhizobium and Brevundimonas increased. In the roots, with the aggravation of wildfire disease, the relative abundance of Legionella and Allorhizobium decreased, and Chryseobacterium, Rhodobacter, and Brevundimonas significantly increased. Compared to healthy samples, the relative abundance of Vermiphilaceae decreased, while Allorhizobium, Azospirillum, Limnobacter, and other genera significantly increased In the hydroponic solution of severely diseased plants.

Considering the recruitment strategies of plants for beneficial microbes, we applied Lefse analysis to determine changes in the bacterial community composition of hydroponic plants under the invasion of wildfire disease in the phyllosphere, roots, and hydroponic solution. We identified enriched bacterial communities in severely diseased phyllosphere, roots, and hydroponic solution for each group using Lefse analysis. Linear Discriminant Analysis (LDA) scores were positively correlated with the significance of each bacterial biomarker in each group (see **Figure 2B**). In comparison, the phyllosphere exhibited a rich diversity of bacterial families, such as Allorhizobium(LDA=4.78), Stenotrophomonas (LDA=4.45), and Enterobacteriaceae (LDA=4.76). Meanwhile, the root system displayed elevated scores for Rhodobacteraceae (LDA=4.46), Flavobacteriales (LDA=5.27), and Chrysebacterium (LDA=5.27). In the hydroponic solution, bacterial enrichment was observed for families such as Chitinophagaceae (LDA=4.45), Azospirillales (LDA=4.47), and Sphingomonadaceae (LDA=4.57).

### 3.2 SourceTracker analysis of bacterial community of health and wildfire-diseased hydroponic plants

**Figure 3:**
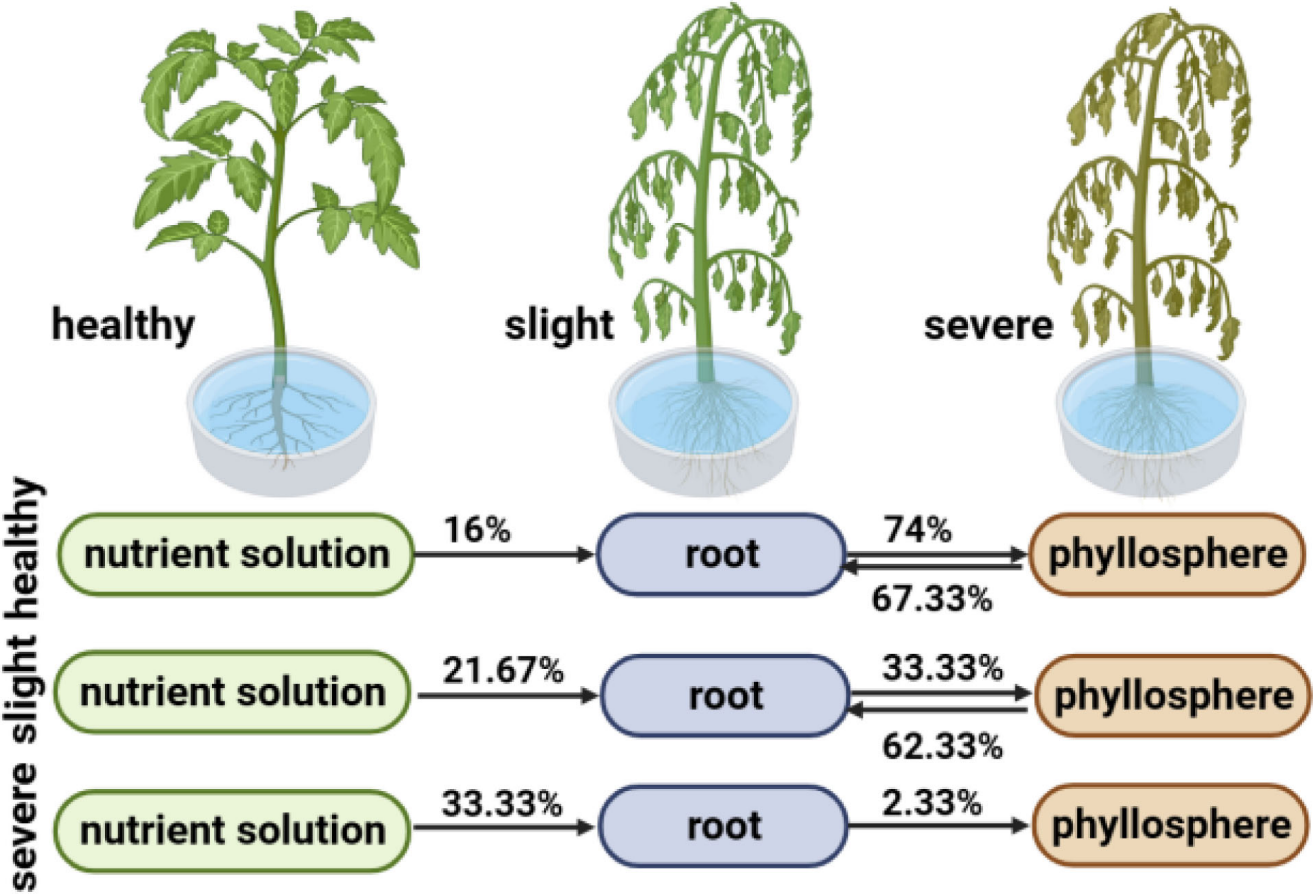
SourceTracker analysis results of healthy, slight infected and severely infected hydroponic plant.

The SourceTracker program was used to study the proportion of endophytic bacterial communities originating from the hydroponic solution. The results revealed differences in microbial sources between healthy and wildfire-diseased hydroponic plants (**Figure 3** and **Supplementary Table 3**). In healthy and mildly infected plants, the majority of the bacterial community in the roots originated from the phyllosphere (H:67.33%, S:62.33%), with a portion originating from the hydroponic solution (H:16.00%, S:23.67%), gradually filtering into the phyllosphere (H:74.00%, S:33.33%). The bacterial communities in the roots and leaves were found to migrate reciprocally. In severely infected plants with wildfire disease, the bacterial community in the roots originated mainly from the hydroponic solution (31.67%). This suggests that the majority of microbial species can be traced back to the plant interior from both the phyllosphere and hydroponic solution.

### 3.3 Molecular ecological network analysis on phyllosphere, root and hydroponic solution communities

Molecular ecological networks (MENs) analyses were used to unravel interactions between phyllosphere, root, and hydroponic solution microbiota in hydroponic Solanaceae crops. The topological characteristics in **Table 1** were consistent with the distinct visualization. The same threshold values were selected for the three compartments: phyllosphere (0.79), root (0.77), and hydroponic solution (0.71). The average connectivity was used to assess network complexity, which decreased progressively from the hydroponic solution (AVEK: 20.86) to the root (AVEK: 7.37) and further to the phyllosphere (AVEK: 4.47). The taxonomic composition of these networks varied among the phyllosphere, root, and hydroponic solution. In the phyllosphere, there were more nodes belonging to Acinetobacter and Stenotrophomonas; while in the root, there were more nodes belonging to Allorhizobium and Sphingobium. In the hydroponic solution, there were more nodes belonging to Novosphingobium and Sphingobium. Additionally, higher modularity and average path distance were observed in the phyllosphere and root compared to the hydroponic solution (see **Supplementary Table 4** and **Supplementary Figure 2**).

**Table 1:** Topological properties of networks in phyllosphere, root and hydroponic solution communities of healthy and infected samples.

Compartment		Vertex	Edge	Average degree	Average path length	Clustering coefficient	Density	Modularity	positive links(%)
L	H	260	570	4.39	3.58	0.04	0.02	0.44	72.98
	S	258	580	4.5	3.55	0.04	0.02	0.45	72.93
	I	219	442	4.03	3.63	0.05	0.02	0.46	72.4
R	H	237	874	7.37	2.84	0.07	0.03	0.32	60.3
	S	234	862	7.37	2.84	0.07	0.03	0.31	60.21
	I	226	814	7.2	2.85	0.06	0.03	0.32	60.44
w	H	309	3155	20.42	2.17	0.11	0.1	0,19	64.63
	S	305	3046	19.97	2.18	0.11	0,1	0.2	64.62
	I	237	2064	17.42	2.18	0.11	0.1	0.19	64.15

**Figure 4:**
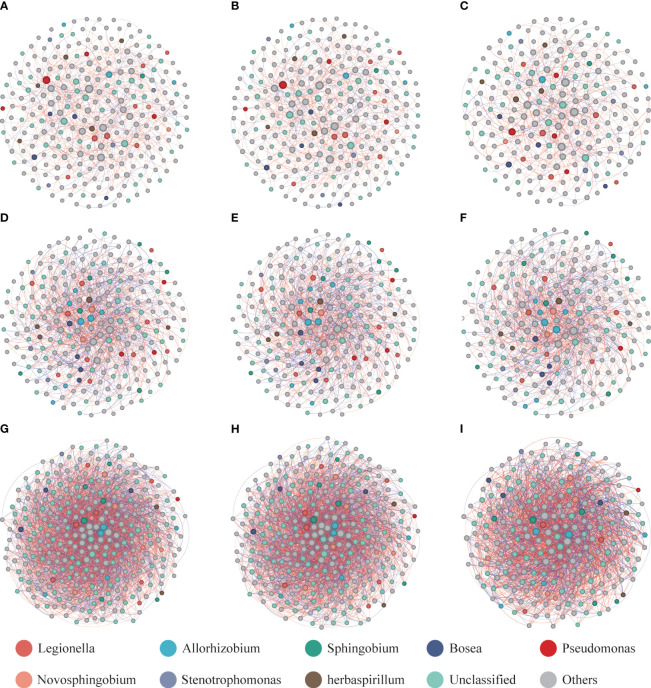
Network visualization of the bacterial communities of healthy and infected hydroponic plants. **(A)** phyllosphere of healthy plant, **(B)** phyllosphere of slight infected plant, **(C)** phyllosphere of severely infected plant, **(D)** root of healthy plant, **(E)** root of slight infected plant, **(F)** root of severely infected plant, **(G)** hydroponic solution of healthy plant, **(H)** hydroponic solution of slight infected plant, and **(I)** hydroponic solution of severely infected plant. A red line indicates a positive interaction between nodes, while a blue line indicates a negative interaction.

To gain a deeper understanding of the interactions among plant phyllosphere, roots, and hydroponic solution microbiota under wildfire disease invasion, the nine networks were visualized, revealing significantly different network structures (**Figure 4**). Our results indicated that during the progression of wildfire disease, the trends in changes in the three compartments were consistent. In the phyllosphere, during the early stages of wildfire disease, there were no significant changes in nodes and links. However, as the disease severity increased, the number of nodes decreased dramatically from 258 to 219, and the links decreased from 580 to 442. In the roots, the network complexity for healthy plants (237 nodes and 874 links) exceeded that of severely diseased plants (226 nodes and 814 links). In the hydroponic solution, with the increasing severity of the disease, the number of nodes decreased from 309 to 305 and then to 237. Similarly, the number of links within the network exhibited a significant declining trend, decreasing from 3155 to 3046 and further to 2064. In summary, our results indicate that as the severity of Pseudomonas infection increases, the networks in different compartments, including the phyllosphere, rhizosphere, and hydroponic solution, are significantly simplified by the disease. Overall, these microbial networks tend to co-occur, and with the increasing wildfire disease severity, the percentage of positive correlation decreases, suggesting a reduction in cooperative relationships among microbial communities in phyllosphere, roots, and hydroponic solution of hydroponic plants.

Interactions between the pathogenic bacterium Pseudomonas and other bacterial members were observed in networks of healthy and wildfire-diseased samples across three different compartments. In both healthy and wildfire-diseased phyllosphere, four nodes of potential pathogenic bacterial OTUs were identified. In the root compartment, three nodes of potentially pathogenic bacteria OTUs were found in both healthy and slightly infected samples, which decreased to one as the disease severity increased. In the hydroponic solution, both healthy and diseased samples had one node for Pseudomonas OTUs. Based on the aforementioned network structures, further analysis of the networks in phyllosphere and roots invaded by pathogenic bacteria was conducted to verify which compartment played a more critical role in the invasion of pathogenic wildfire disease. In the network of mildly diseased phyllosphere, the number of links between potential pathogenic Pseudomonas OTUs and other microbial members (total of 30 links) was higher than in severely diseased plants (22 links). Similarly, In the network of mildly diseased roots, the number of links between potential pathogenic Pseudomonas OTUs and other microbial members (15 links) was higher than in severely diseased plants (5 links). This indicates that the invasion of pathogenic bacteria involves more interactions with other microbial members in the phyllosphere and root of slight infected plants.

### 3.4 Network of interactions among potential pathogenic *Pseudomonas* and other microbial members in slightly infected phyllosphere and root

**Figure 5:**
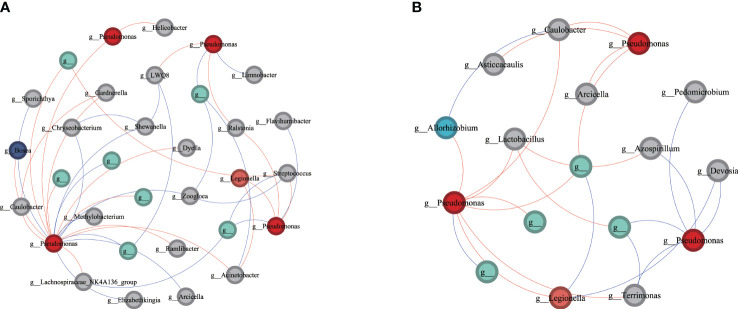
Network of interactions between the pathogen (Pseudomonas) and other bacteria species in bacteria communities of slightly infected **(A)** phyllosphere and **(B)** root. Each node is labeled at the genus level and unclassified OTUs are not labeled. A red line indicates a positive interaction between nodes, while a blue line indicates a negative interaction.

To further elucidate which compartments microbes may play a crucial role in aiding or inhibiting wildfire disease, we analyzed subnetworks of interactions between the pathogenic Pseudomonas and other microbial members to identify the “inferred” key microbes in infected phyllosphere and root networks (see **Figure 5**). In the subnetwork of slightly infected phyllosphere, the pathogenic Pseudomonas showed positive correlations with genera such as *Ralstonia, Legionella, Bosea, and Dyella*, while exhibiting negative correlations with genera like *Chryseobacterium, Limnobacter, Methylobacterium, Zoogloea*, and *Arcicella*. In the subnetwork of mildly infected roots, the pathogenic *Pseudomonas* displayed positive correlations with *Arcicella, Caulobacter, Allorhizobium*, and negative correlations with genera like *Terrimonas, Azospirillum, Devosia*, and *Pedomicrobium*. These bacteria, positively or negatively correlated with the potential pathogenic *Pseudomonas*, may play crucial roles in assisting or inhibiting bacterial wilt infection. Our observations suggest that during the invasion of wildfire disease, the pathogenic *Pseudomonas* may receive assistance from locally correlated microbial members while being inhibited by some negatively correlated taxa, engaging in resource competition. Particularly noteworthy is *Caulobacter*, which exhibits a negative correlation with both pathogenic bacteria in the roots.

## 4 Discussion

Plant disease is traditionally considered as the product of the ‘disease triangle’: a susceptible host, a virulent pathogen, and an abiotic environment conducive to infection (Lucas, 2009). During plant disease invasion, the structural and assembly of plant-associated microbial communities are essential for advancing the co-evolutionary theory of plant-microbiome interactions (Tian et al., 2020). In this study, we linked the bacterial communities in the phyllosphere, root, and hydroponic solution of Solanaceae crops to the severity of bacterial wilt, gaining insights into the impact of bacterial wilt on these three compartments. Our results indicate that the microbial communities in all three compartments are simultaneously influenced by niches and disease. Pseudomonas infection is closely associated with the diversity and microbial interactions of the plant bacterial community. However, the microbial communities in the roots and phyllosphere exhibit greater sensitivity to plant disease compared to the microbial community in the hydroponic solution.

Our results indicate that, regardless of the plant’s health status, distinct microbial communities form in the phyllosphere, root, and hydroponic solution. This suggests that plant compartments have a greater impact on the distribution of microbial communities than diseases and other environmental factors. This aligns with findings from studies on pepper with Fusarium wilt disease (Gao et al., 2021) and wheat under different fertilization regimes (Xiong et al., 2021). The pattern of microbial diversity (Shannon index) displays a clear gradient from the phyllosphere to the root and then to the hydroponic solution. All of these observations suggest that ecological niches play a dominant role in shaping the environmental factors governing the formation of plant microbial communities. We note that in the hydroponically grown plant, the abundance of Proteobacteria and Firmicutes is higher in both the phyllosphere and roots. This is similar to the composition observed in other hydroponically cultivated plants, such as lettuce, where Proteobacteria dominates the phyllosphere (Kyere et al., 2020), and in the roots of hydroponically grown cereal potatoes and wheat, where the dominant phyla are Proteobacteria and Firmicutes (Sheridan et al., 2017). Therefore, we hypothesize that the distribution of different bacterial phyla associated with the leaves and roots of hydroponically grown plants is likely similar across most hydrophytic plants.

We observed differential impacts of wildfire disease on the bacterial communities in the phyllosphere, roots, and hydroponic solution, with the phyllosphere and roots being more sensitive to the disease. Our results indicate that, during the early stages of pathogen invasion, the abundance of the pathogen is relatively low, exerting minimal impact on the bacterial community. The diversity of microbial communities in both the phyllosphere and roots remains relatively stable. However, as the infection intensifies and the plant’s defense systems are compromised, the microbial communities in both the leaves and roots undergo an imbalance, leading to a significant decrease in community diversity. This aligns with previous findings (Tao et al., 2021), where the diversity of plant phyllosphere surface microbiota decreased with the progression of phyllosphere wildfire disease.

The analysis of bacterial communities in plants affected by wildfire disease through taxonomic classification reveals the impact of Pseudomonas infection on different plant compartments. We observed a low abundance of potential pathogenic Pseudomonas OTUs in all healthy samples, and We cannot technically confirm that all OTUs assigned to Pseudomonas are pathogenic just by ITS sequences. However, with the progression of disease severity, accompanied by changes in the abundance of other bacteria. In the phyllosphere, the abundance of Allorhizobium and Brevundimonas rapidly increases with disease severity, while Chryseobacterium significantly enriches in the infected roots. These compositional changes may be outcomes of pathogen invasion. Root bacteria form symbiotic relationships with plants, creating nodules to fix atmospheric nitrogen into plant-absorbable compounds. This enhances the plant’s survival under environmental stress by improving soil conditions and degrading toxic substances (Siddiqui and Shaukat, 2002; Kuzmanović et al., 2022). Previous studies (Gutiérrez Mañero et al., 2003; Kumar et al., 2021) have identified Chryseobacterium as a Plant Growth-Promoting (PGP) bacterium, promoting seed germination and influencing root total nitrogen content and biological nitrogen fixation, and protecting plants from pathogenic invasion. Research by VOGEL et al. (Vogel et al., 2012; Asaf et al., 2020) indicated that Sphingomonas, a Gram-negative bacterium, produced plant hormones, protected plants through substrate competition against Pseudomonas syringae (wildfire pathogen) and various pathogenic fungi, and promoted plant growth under stressful conditions. LDA analysis demonstrates the enrichment of these three bacteria in the phyllosphere, roots, and hydroponic solution of severely diseased plants, suggesting a “call for help” strategy by plants under biotic stress (pathogen invasion). Pathogen invasion induces changes in the plant’s immune system (Jones and Dangl, 2006), altering secretions to recruit beneficial microorganisms from the environment. This interaction, through competition or modulation of plant defense responses, endows the roots and leaves with the ability to resist wildfire disease. These results indicate that plant niches may create distinct ecological niches for specific microbial communities. When invaded by pathogens, plants demonstrate the ability to recognize signal molecules and adapt their immune systems in each niche (Cook et al., 2015). The alterations in secretions recruit beneficial microorganisms, enabling the roots and leaves to develop resistance against wildfire disease.

Understanding the potential sources of crop-associated microbial communities provides a roadmap for the process of pathogenic invasion. Our study found that in healthy and slightly infected plants, the bacterial communities of hydroponically grown crops mainly originate from the phyllosphere. In severely diseased plants, the microbial sources differ, with the root bacterial community primarily originating from the hydroponic solution. This contrasts with previous study results where the soil was identified as the primary source of microbial species in plant root ecosystems, gradually occupying different niches (Lareen et al., 2016).For hydroponically grown plants, the main source of microbial communities is the plant phyllosphere. Therefore, the invasion of pathogenic bacteria may initiate from the phyllosphere, proliferating and subsequently transferring to the plant roots. In severely diseased plants, compared to healthy plants, the disruption of the plant’s vascular system responsible for material transport in the phyllosphere (Sohrabi et al., 2023) implies higher proportions of unknown and hydroponic solution-derived microbial communities in the roots. This indicates that disease damage has altered the potential source pathways of microbial communities in hydroponically grown plants, disrupting material transport between the phyllosphere and roots.

Numerous studies have already revealed the cooperative and competitive interactions among microorganisms, as well as how network modularity can influence community stability (Faust and Raes, 2012; Coyte et al., 2015). However, it remains unclear how the interactions between pathogenic bacteria and bacterial communities in different plant compartments change under varying degrees of infection. In addition to analyzing changes in the composition of microbial communities in Solanaceae crops, the symbiotic network further illustrates the interactions between communities in different plant compartments, providing valuable information on the impact of microbial community changes on plant health. Our study reveals that networks in Solanaceae crops are significantly influenced by disease and plant compartments, with disease reducing network complexity. The network composition of the phyllosphere, roots, and hydroponic solutions differs, with hydroponic solutions having the most complex network. However, the patterns of change in phyllosphere, root, and hydroponic networks are similar. As the severity of pathogen invasion increases, network links and average degrees gradually decrease, and positive percentages decrease. This indicates that Pseudomonas syringae infection disrupts the existing interaction relationships between microbial communities and has irreversible effects. This is consistent with previous research findings that Pseudomonas syringae invasion results in the occupation of abundant resources on phyllosphere surfaces and roots, forcing other bacteria to develop negative interactions with Pseudomonas syringae in later stages to compete for resources (Alba et al., 2017). This competition relationship contributes to improving the stability of the microbial community

Additionally, we observed that in the network of slightly diseased phyllosphere surfaces, the number of connections between potential pathogenic Pseudomonas OTUs and other microbial members is 30, whereas in the case of severe disease, it is 22. In the network of mildly diseased root systems, the connections between potential pathogenic Pseudomonas OTUs and other microbial members amount to 15, while in the case of severe disease, it decreases to 5. Based on these findings and the results of our traceback analysis, we determine that in severely diseased plants, both the pathways from phyllosphere to roots and from roots to phyllosphere have been disrupted. Consequently, in mildly diseased plants, the interaction between Pseudomonas and other microbial members appears to be more intimate. Both the phyllosphere surfaces and root systems of the plants play crucial roles as migration hubs during the progression of bacterial wildfire.

The network analysis also reveals the relationships between the pathogen and other relevant bacterial species (**Figure 5**). Plant pathogens interact with other bacteria in both cooperative (negative) and competitive (positive) ways, and microorganisms with positive or negative connections to the pathogen are considered “pathogen antagonists” (Raghavendra and Newcombe, 2013) or “pathogen promoters” (Ridout and Newcombe, 2015), respectively. Therefore, Allorhizobium, Caulobacter, Dosea, and Ralstonia may act as pathogen antagonists, while Chryseobacterium, Azospirillum, Devosia, and Pedomicrobium may act as pathogen promoters. It is noteworthy that the potential “pathogen antagonist” Caulobacter, which shows a strong negative correlation with the pathogen Pseudomonas in diseased roots, may play a crucial role in inhibiting the growth of the pathogen. We found that beneficial bacteria, such as Chryseobacterium, Azospirillum, Devosia (Fibach-Paldi et al., 2012; Singh et al., 2013;53), closely collaborate with the pathogen. Through our source tracking results, it can be inferred that beneficial bacteria lead to the proliferation of bacteria on the phyllosphere and in the roots. They circulate mutually in phyllosphere and roots, triggering the outbreak of wildfire disease. This is consistent with previous research results (Lamichhane and Venturi, 2015) suggesting that harmless or beneficial bacteria may cause the invasion and proliferation of the pathogen. It has been demonstrated that pathogens do not operate independently, but their virulence is mediated by their interaction with other pathogens (Denny, 2006; Purahong et al., 2018). Ralstonia is a common plant pathogen (Luo et al., 2019), and the positive interaction between Ralstonia and Pseudomonas may be because Ralstonia also needs to reproduce by competing for substrates, thus inhibiting the growth of the wildfire disease pathogen Pseudomonas and decreasing the severity and development of wildfire disease. Caulobacter is a Gram-negative bacterium that can be isolated from various plant species such as Arabidopsis, watermelon, and maize (Yang et al., 2020; Berrios, 2021). Research (Luo et al., 2019) has shown that Caulobacter parasitizes both the roots and aboveground parts of Arabidopsis. It not only promotes lateral root formation and increases phyllosphere number and size but also protects plants from external stress and disease invasion (Carter, 2021). Currently, there is limited research on the antagonistic mechanism of Caulobacter against plant pathogens. Future studies should focus on the potential interactions between Caulobacter and pathogens.

## 5 Conclusions

Our study revealed significant differences in bacterial communities on the phyllosphere surface, roots, and hydroponic solution under varying degrees of wildfire disease conditions. The impact of host compartments on the assembly of bacterial microbiota was most pronounced, followed by the wildfire disease itself, with the phyllosphere and roots being more affected than the hydroponic solution. The invasion of the pathogen altered the composition of the plant’s microbial community, resulting in reduced bacterial community diversity and network complexity. We also identified that the invasion of wildfire disease initiated from the phyllosphere surface. Chryseobacterium, Azospirillum, and Devosia may contribute to the severity and progression of wildfire disease, while Caulobacter might play a significant role in suppressing wildfire disease.

## Data Availability

The original contributions presented in the study are included in the article/**Supplementary Material**, further inquiries can be directed to the corresponding author/s.
