# Supplementary material for: Role of bacterial pathogens in microbial ecological networks in hydroponic plants
Source: Front Plant Sci. 2024 Sep 3;15:1403226. doi: 10.3389/fpls.2024.1403226 (PMC11405252; doi:10.3389/fpls.2024.1403226)
Supplement: Supplementary file 1 [file DataSheet1.docx]

Supplementary Material

Role of bacterial pathogens in microbial ecological networks in hydroponic plants

Wenyi Liu1,4#, Zhihua Zhang2#, Bin Zhang3#, Yi Zhu2, Congwen Zhu2, Chaoyong Chen2, Fangxu Zhang2, Feng Liu2, Jixiang Ai2, Wei Wang2, Wuyuan Kong2, Haoming Xiang2, Weifeng Wang2, Daoxin Gong1, Delong Meng3*, and Li Zhu2*

*** Correspondence:** Li Zhu:[cdyc202112@163.com](mailto:cdyc202112@163.com;);Delong Meng:delong.meng@csu.edu.cn


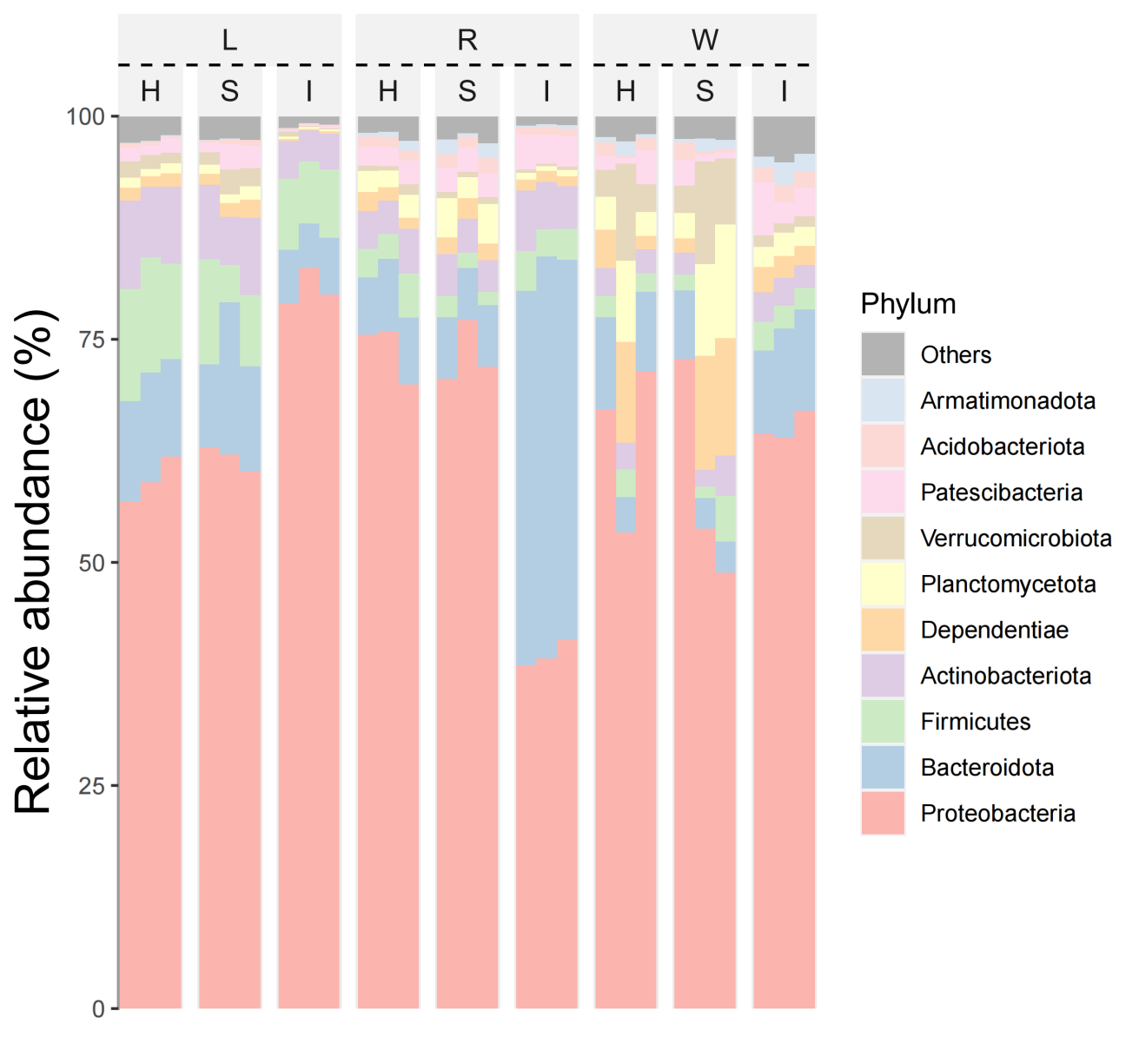


**Supplementary Figure 1.** Relative abundance of bacterial phylum of top 10 most dominant OTUs in healthy and infected phyllosphere, root and hydroponic solution.


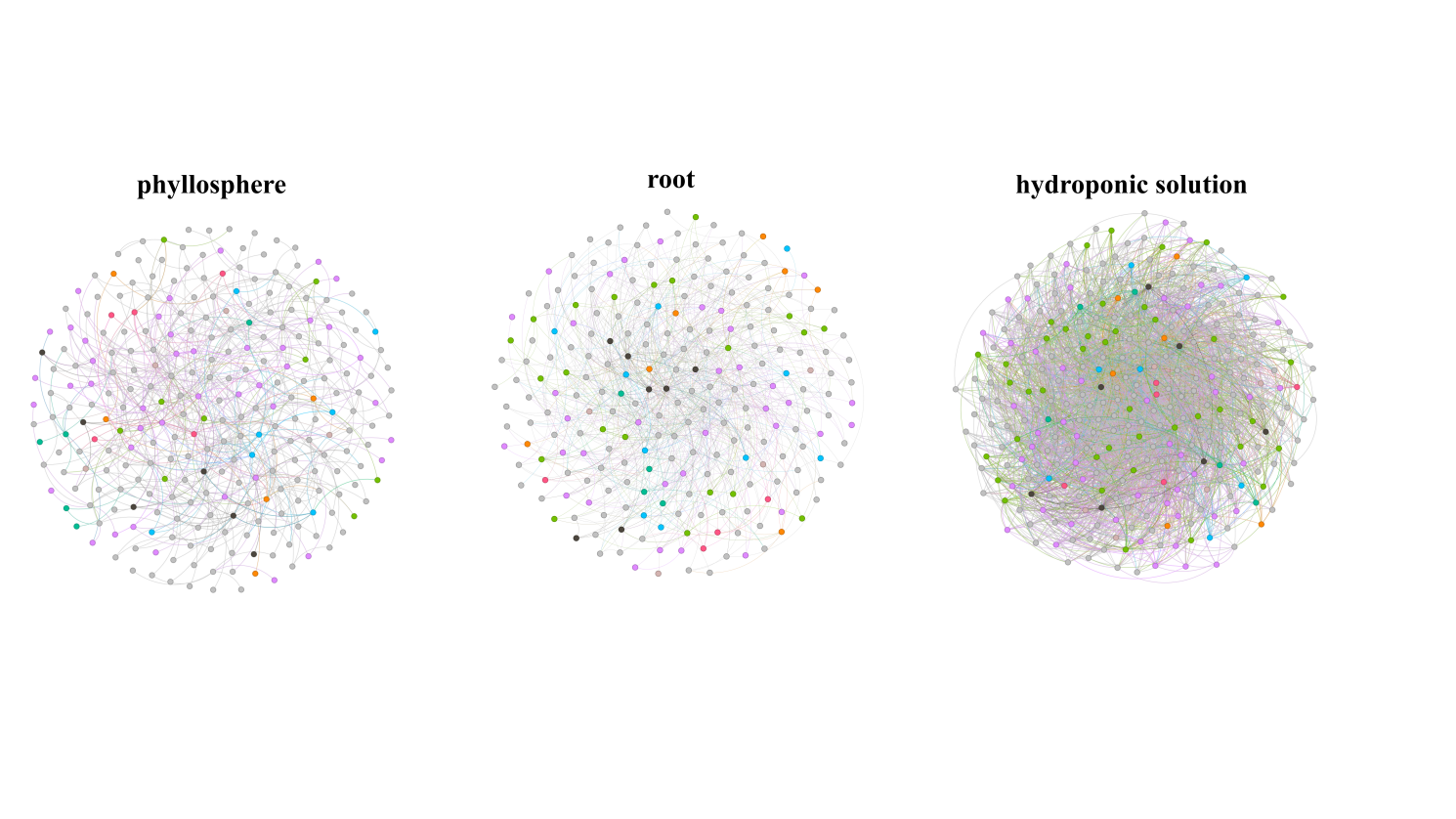


**Supplementary Figure 2.**Topological properties of networks in phyllosphere, root and hydroponic solution communities.

**Supplementary Table 1.** Effects of plant compartment and disease on bacterial community structure based on PERMANOVA.

| Variables | Df | SumOfSqs | R2 | F | Pr(>F) |
| --- | --- | --- | --- | --- | --- |
| Compartment | 2 | 2.6824813 | 0.38974178 | 8.365928 | 0.001 |
| Degree of disease | 2 | 0.6731532 | 0.09780345 | 2.099381 | 0.02 |

**Supplementary Table 2.** Effects of plant compartment on bacterial community structure based on PERMANOVA.

| Compartment | SumOfSqs | R^2^ | F | Pr(>F) |
| --- | --- | --- | --- | --- |
| L | 1.11 | 0.76 | 9.73 | 0.02 |
| R | 0.69 | 0.72 | 7.74 | 0.006 |
| W | 0.86 | 0.48 | 2.81 | 0.02 |

**Supplementary Table 3.**The percentage contributions and standard deviations of potential sources for bacteria in phyllosphere, root and hydroponic solution communities in the distribution system calculated by SourceTracker.

| Sample ID | phyllosphere | root | hydroponic solution | unknown |  |
| --- | --- | --- | --- | --- | --- |
|  |  |  |  |  |  |
| HL1 | 0 | 0.72 | 0.01 | 0.27 |  |
| HL2 | 0 | 0.74 | 0.01 | 0.23 |  |
| HL3 | 0 | 0.73 | 0.01 | 0.26 |  |
| HR1 | 0.67 | 0 | 0.17 | 0.16 |  |
| HR2 | 0.66 | 0 | 0.18 | 0.16 |  |
| HR3 | 0.69 | 0 | 0.13 | 0.18 |  |
| SL1 | 0 | 0.18 | 0.01 | 0.82 |  |
| SL2 | 0 | 0.45 | 0.01 | 0.54 |  |
| SL3 | 0 | 0.37 | 0.01 | 0.62 |  |
| SR1 | 0.56 | 0 | 0.25 | 0.19 |  |
| SR2 | 0.77 | 0 | 0.12 | 0.12 |  |
| SR3 | 0.54 | 0 | 0.28 | 0.18 |  |
| IL1 | 0 | 0.03 | 0.01 | 0.96 |  |
| IL2 | 0 | 0.02 | 0.01 | 0.98 |  |
| IL3 | 0 | 0.02 | 0.01 | 0.98 |  |
| IR1 | 0.01 | 0 | 0.3 | 0.69 |  |
| IR2 | 0.01 | 0 | 0.3 | 0.69 |  |
| IR3 | 0.01 | 0 | 0.35 | 0.64 |  |
